# App-Based Relaxation Exercises for Patients With Chronic Neck Pain: Pragmatic Randomized Trial

**DOI:** 10.2196/31482

**Published:** 2022-01-07

**Authors:** Daniel Pach, Susanne Blödt, Jiani Wang, Theresa Keller, Beatrice Bergmann, Alizé A Rogge, Jürgen Barth, Katja Icke, Stephanie Roll, Claudia M Witt

**Affiliations:** 1 Institute of Social Medicine, Epidemiology and Health Economics Charité – Universitätsmedizin Berlin, corporate member of Freie Universität Berlin and Humboldt-Universität zu Berlin Berlin Germany; 2 Institute for Complementary and Integrative Medicine University Hospital Zurich and University of Zurich Zurich Switzerland; 3 Institute of Biometry and Clinical Epidemiology Charité – Universitätsmedizin Berlin, corporate member of Freie Universität Berlin and Humboldt- Universität zu Berlin Berlin Germany; 4 Center for Integrative Medicine University of Maryland School of Medicine Baltimore, MD United States

**Keywords:** neck pain, relaxation, RCT, mHealth, smartphone app, mobile phone

## Abstract

**Background:**

Chronic neck pain is a highly prevalent condition. Learning a relaxation technique is recommended by numerous guidelines for chronic neck pain. Smartphone apps can provide relaxation exercises; however, their effectiveness, especially in a self-care setting, is unclear.

**Objective:**

The aim of this pragmatic randomized trial is to evaluate whether app-based relaxation exercises, including audio-based autogenic training, mindfulness meditation, or guided imagery, are more effective in reducing chronic neck pain than usual care alone.

**Methods:**

Smartphone owners aged 18 to 65 years with chronic (>12 weeks) neck pain and the previous week’s average neck pain intensity ≥4 on the Numeric Rating Scale (0=no pain to 10=worst possible pain) were randomized into either an intervention group to practice app-based relaxation exercises or a control group (usual care and app for data entry only). For both groups, the follow-up data were collected using app-based diaries and questionnaires. The primary outcome was the mean neck pain intensity during the first 3 months based on daily measurements. Secondary outcomes included neck pain based on weekly measurements, pain acceptance, neck pain–related stress, sick-leave days, pain medication intake, and adherence, which were all measured until the 6-month follow-up. For the primary analysis, analysis of covariance adjusted for baseline neck pain intensity was used.

**Results:**

We screened 748 participants and enrolled 220 participants (mean age 38.9, SD 11.3 years; mean baseline neck pain 5.7, SD 1.3 points). The mean neck pain intensity in both groups decreased over 3 months; however, no statistically significant difference between the groups was found (intervention: 4.1 points, 95% CI 3.8-4.4; control: 3.8 points, 95% CI 3.5-4.1; group difference: 0.3 points, 95% CI −0.2 to 0.7; *P*=.23). In addition, no statistically significant between-group differences regarding neck pain intensity after 6 months, responder rate, pain acceptance, pain medication intake, or sick-leave days were observed. There were no serious adverse events that were considered related to the trial intervention. In week 12, only 40% (44/110) of the participants in the intervention group continued to practice the exercises with the app.

**Conclusions:**

The study app did not effectively reduce chronic neck pain or keep the participants engaged in exercising in a self-care setting. Future studies on app-based relaxation interventions should take into account the most recent scientific findings for behavior change techniques.

**Trial Registration:**

ClinicalTrials.gov NCT02019134; https://clinicaltrials.gov/ct2/show/NCT02019134

**International Registered Report Identifier (IRRID):**

RR2-10.1186/1745-6215-15-490

## Introduction

Neck pain is a global public health issue entailing a high socioeconomic burden [1,2]; moreover, it is one of the top 5 global chronic pain conditions in terms of prevalence and cause of disability [3,4]. According to the data from the European Social Survey 2014 [5], approximately 40% of all respondents reported back or neck pain. These results indicated the highest prevalence of back or neck pain in Germany (54.05%).

In most cases, neck pain is nonspecific [1]. Hence, the treatment is complex and costly. Pharmacological approaches are often used to alleviate chronic pain; however, these approaches include possible risks of tolerance, dependence, and addiction when using opioids [6,7]. Moreover, previous research showed that exercise treatment might also be beneficial in patients with neck pain [3].

Mind–body therapies focus on the interactions among the brain, mind, body, and behavior and their effects on health and disease [8]. As components of mind–body medicine, relaxation techniques have gained wide acceptance within conventional medicine [9]. The relaxation response leads to a variety of physiological benefits that may enhance pain relief through reduced sympathetic activity, decreased muscular tension, modulated pain awareness, and increased release of endogenous opioids [10,11]. Studies directly comparing the effects of self-administered versus therapist-administered interventions found similar effects on pain reduction [12]. Moreover, according to the recent *Neck Pain Guideline* of the German Society of General Practice and Family Medicine [13], learning a relaxation technique is recommended for patients with nonspecific chronic neck pain that lasts for >12 weeks. Thus, relaxation techniques alone or in addition to conventional medical care can influence the treatment and rehabilitation of chronic neck pain. However, the accessibility of cognitive and mind–body therapies for chronic low back pain and neck pain remains a major challenge [14].

Medical smartphone apps or other mobile digital health solutions can allow easy access to self-care activities [15] and support behavior changes by incorporating features such as the provision of information, tracking of activity, or providing feedback. A review [16] identified 606 mindfulness apps; however, only 3.8% (23/606) of those apps actually provided mindfulness training, and only 1 app [17] was evaluated in a randomized controlled trial (RCT). Another review [8] on apps with self-management support functions for people with persistent pain identified only 2 evidence-based apps; however, none of them were for chronic pain.

In this study, we aim to conduct a pragmatic app-based RCT to evaluate whether app-based audio relaxation exercises are more effective in reducing chronic neck pain than usual care.

## Methods

### Study Design

The trial design and methods have been published elsewhere [18] and have not been changed afterward. The study app remained *frozen* without any updates during the trial.

**Figure 1:**
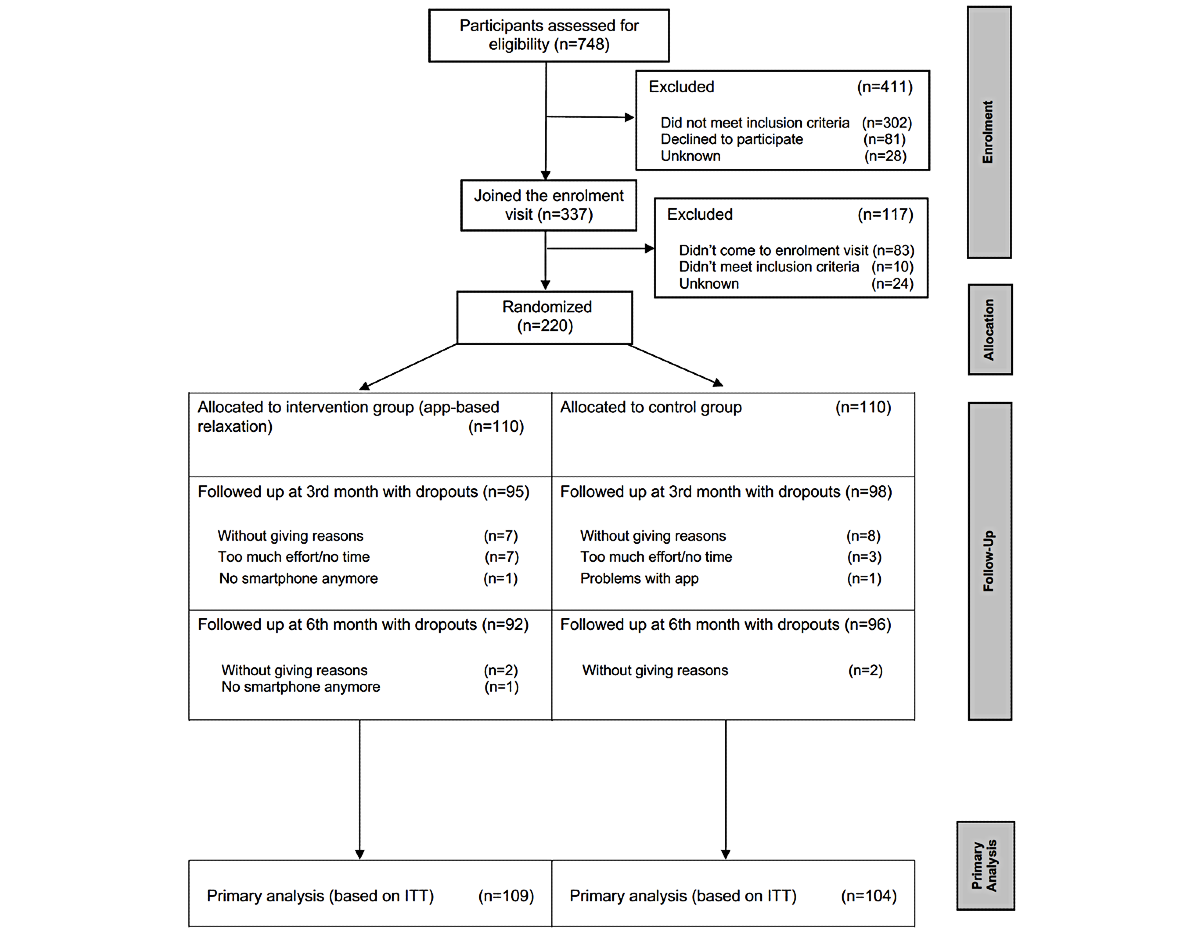
Trial flow chart. ITT: intention-to-treat.

We conducted a 2-armed, randomized, parallel-group, single-center pragmatic trial to investigate the effectiveness of additional relaxation exercises delivered by a smartphone app compared with usual care alone. Participants were randomized in a 1:1 ratio to either the app-based relaxation intervention group or the control group. The trial flow is presented in Figure 1.

The intervention duration was 6 months, with the primary outcome summarizing the effect of the first 3 months.

### Participants and Setting

The first participant was randomized on March 31, 2014, and the final data recording was on January 11, 2017, in Berlin, Germany. Information on the study was posted with brochures and posters in universities, gyms, and general practitioners’ offices. Moreover, the study was advertised in local subways from December 2014 to July 2015. Eligibility was checked by a study nurse at the study site. Eligible participants completed the paper-and-pencil baseline questionnaires. Then, the study nurse helped the participants install the app on their own smartphones and provided a randomly allocated code to activate the study app and the respective app features according to the group allocation. Participants received compensation of €20 (US $ 22.60) after participating in the study.

The inclusion criteria were as follows: aged 18-65 years, chronic neck pain within at least the past 12 weeks, average neck pain intensity ≥4 on the Numeric Rating Scale (NRS; 0=no pain to 10=worst possible pain) in the previous week, possession of a smartphone (iOS or Android), willingness to be randomized and follow the app-delivered interventions, and willingness to enter data through the study app.

Participants were excluded if their neck pain was caused by a known malignant disease, trauma, the presence of a known rheumatic disorder, a history or planned surgery of the spinal column of the lower neck in the next 6 months, known neurological symptoms (eg, radicular symptoms because of a prolapsed disk), regular intake of analgesics (more than once per week) because of additional disease, intake of centrally acting analgesics, or a history of severe acute or chronic disorders that did not allow participation in the study.

Further exclusion criteria were known alcohol or substance abuse, insufficient German language skills, current application for a pension claim, participation in another clinical trial during the 6 months before the study and parallel to the study, applying regular relaxation techniques, mindfulness meditation, or any other *mindfulness-based* therapy 6 weeks before the study or planned in the next 6 months.

Participants in both groups were allowed to continue with their usual care (medical and nonmedical); however, the regular application of any other relaxation techniques, including mindfulness meditation or mindfulness-based training, was not permitted.

The follow-up data (daily, weekly, and at the third and sixth month) were collected through the app-based questionnaires and by in-app tracking of the length of the practiced exercises. Serious adverse events were documented during the study period to evaluate safety.

### The Relaxneck App

#### Overview

The study app *Relaxneck* was developed by the Institute of Social Medicine, Epidemiology and Health Economics, Charité–Universitätsmedizin Berlin, Germany, together with Smart Mobile Factory, Berlin, Germany, which is an agency focused on mobile solutions [18]. The app supported iOS and Android systems and was available in the German Apple Appstore and the Google Play Store free of charge. However, the app could only be activated by entering an individual code assigned to each study participant by the study nurse.

**Figure 2:**
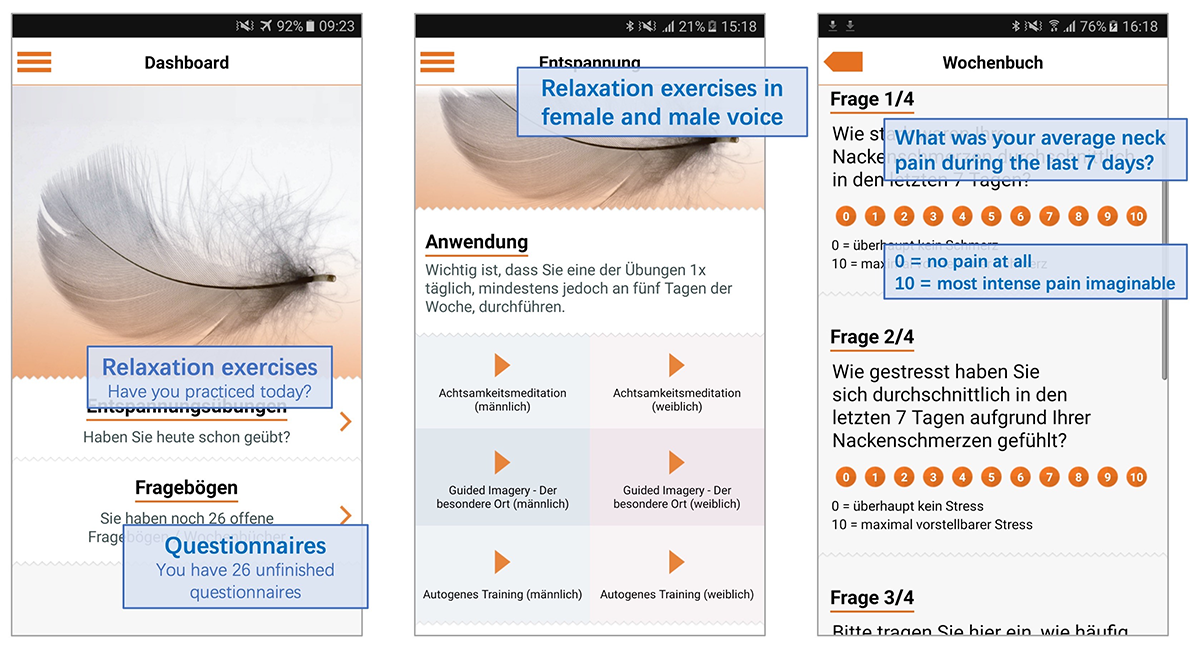
Screenshots of the study app (dashboard, relaxation exercises, and questionnaires).

The app supported notification features, a diary, and questionnaire options for all participants, whereas it provided audio relaxation exercises only for those in the intervention group. The app’s user interface and content were available in the German language (Figure 2). The app concept was approved by the data protection officer of the Charité–Universitätsmedizin Berlin.

#### App-Based Relaxation Interventions

##### Overview

The duration of the audios for the relaxation interventions, as well as their intensity and dosage; the use of push notifications; the diary content: and the German translation of guided imagery instructions resulted from stakeholder engagement during the planning phase of the study [18].

There were 3 types of exercises (autogenic training, mindfulness meditation, and guided imagery), with a length of 15 minutes each, that were available in 2 versions (female and male voices) in the study app for the intervention group. They were accompanied by a short instructional text (Figure 2). Relaxation exercises could be applied in different positions (sitting, walking, and lying) according to the participants’ needs. It was recommended to apply a relaxation exercise daily or at least 5 days per week for 6 months.

##### Autogenic Training

Autogenic training is a form of self-relaxation technique that is commonly used to treat stress disorders, pain, and anxiety [19-21]. Autogenic training was developed by the German psychiatrist Johannes Schultz in 1932. It focuses on the physical sensation of the breath or heartbeat and visualizes the body as warm, heavy, or relaxed [21]. Participants learn to react to 6 verbal commands, such as “my arms are very heavy,” “my heart beats regularly and calm,” and “my belly is warm,” to make the body feel relaxed [18].

##### Mindfulness Meditation

Mindfulness is a practice based on Vipassana (ie, insight) meditation, which has Buddhist roots. It is defined as “paying attention in a particular way: on purpose, in the present moment and in a nonjudgmental way” [22]. It focuses on the breath and uses it as an anchor when the mind starts to wander [18]. This concept is also used in mindfulness-based stress reduction developed by Kabat-Zinn [22-24].

##### Guided Imagery

In guided imagery, the mind is directed to intentionally create images to produce positive changes [25]. The audio guides the participants to visualize or conjure a place that is associated with positive feelings such as safety, security, and well-being. The guided imagery audio is accompanied by soft background music and directs visualization and imagination to a pleasant and peaceful place that has meaning for the participant to replace negative or stressful feelings [26].

#### Behavior Change Techniques in the App

To enhance changes in participants’ behavior, behavior change techniques (BCTs) can be implemented in intervention settings [27]. As this was not a common feature in app development in 2013, we retrospectively analyzed the Relaxneck app using the BCT taxonomy (version 1) by Michie et al [27] to identify BCTs that were represented in the app, although not formally preplanned.

### App for the Control Group

Participants in the control group downloaded the same app as the intervention group. All study data after baseline measurements were collected by means of app-based diaries and questionnaires. The participants were able to activate reminders for the questionnaire notifications. However, no intervention features, that is, relaxation exercises, were accessible in their version of the app. The relaxation exercises were activated after 6 months after all the survey data were collected. In addition, participants could continue using usual care, defined as all medical and nonmedical treatments, while using the app; however, relaxation techniques, mindfulness meditation, or any other mindfulness-based trainings were not permitted to be practiced during the study.

### Outcome Measurements

The primary outcome measure was the mean neck pain intensity during the first 3 months of intervention based on daily measurements of pain intensity on the NRS (0=no pain to 10=worst possible pain) [18].

The secondary outcome parameters included the mean pain intensity during the first 6 months after randomization based on daily measurements, the mean pain intensity measured weekly (using NRS) as the average pain intensity of the previous 7 days over 3 and 6 months, pain acceptance (German version of Chronic Pain Acceptance Questionnaire [28]), *neck pain–related stress*, sick-leave days, and pain medication intake. Data on adherence, self-reported general changes in neck pain, suspected adverse reactions, and serious adverse events were additionally collected [18].

If a weekly survey had not been completed, the patient received an SMS text message as a reminder; if 2 consecutive weekly surveys had not been completed, the patient was contacted by telephone call; if there was no response after 2 calls, the patient received a reminder letter.

The number of participants who practiced the exercises was recorded to reflect exercise adherence over time. Practice of the exercise was defined by (1) tracking the number (and duration) of applied types of intervention with the app and (2) asking the participants weekly about the number of applied types of intervention without using the app. The complete stop of filling in any data with the study app was defined as participant dropout. Adverse events and suspected adverse reactions (only in the intervention group) were assessed after 3 and 6 months.

### Sample Size

According to previous literature [29], an effect size of 0.62 has been described for mind–body therapies compared with no intervention in a group setting. We assumed a smaller effect size of 0.4 (Cohen *d*, baseline adjusted) for individual self-care relaxation exercise compared with usual care alone, as individuals might be less focused and consequently less adherent in a self-care setting [18]. To obtain a power of 80% using a 2-sided *t* test with a significance level of .05, 100 participants for each treatment group were needed (a total of 200 participants). Thus, a final sample size of 110 participants per group (220 in total), allowing a dropout rate of 9.1%, was required.

### Randomization, Allocation, and Implementation

Eligible participants were randomized to either the intervention (app-based relaxation and usual care) or the control (usual care only) group using blocked randomization with variable block lengths and an allocation ratio of 1:1, that is, 110:110 participants. The randomization sequence was generated by a data manager who was not involved in the analysis of the data or the enrollment of the patients; SAS (version 9.3, SAS Inc) was used for this process. The randomization list was included in a safe Microsoft Access database to ensure that it was not accessible during the randomization process of individual participants and that the screened patients were strictly consecutively enrolled. The randomization process was conducted by the study office at the Institute of Social Medicine, Epidemiology and Health Economics. To ensure allocation concealment, first, the study team added the participants’ information into the database, and then, random allocation of the participants into the intervention or control group was performed.

### Statistical Analysis

For the primary analysis of the primary outcome (mean pain intensity over 3 months measured as the daily pain intensity), an analysis of covariance with a fixed factor of *treatment group*, adjusted for the baseline NRS value (fixed covariate), was performed. The analysis was based on the full analysis set (all available data without imputation of missing values, as only a small number of missing values was expected based on experiences with a previous app-based study conducted by our study team in a similar study setting [30]) based on the intention-to-treat principle with a 2-sided significance level of .05.

All the secondary analyses were explorative, and *P* values were interpreted as such. The secondary outcomes were analyzed for the full analysis set, similar to the primary analysis, depending on the scale and distribution of the outcome, that is, analysis of covariance or logistic regression, adjusted for the respective baseline value. For sensitivity analysis, the primary analysis of the primary outcome was repeated based on the per-protocol population.

Subgroup analyses were performed on the primary outcome by including an interaction term (subgroup variable by treatment) in the main model and performing separate analyses for each subgroup. Subgroups were specified with covariates in age, education (>10 years of school education or ≤10 years of school education), sex (male or female), and duration of disease. Kaplan–Meier survival analysis was conducted to investigate whether the app features (with or without app-based intervention content) predicted the dropout of app use.

SAS version 9.4 (SAS Inc) was used for data analysis, except for the Kaplan–Meier survival analysis for adherence, which was conducted using SPSS version 22.0 (SPSS Inc).

### Ethics

The study was approved by the local ethics review board at the Charité–Universitätsmedizin, Berlin (approval number Relaxneck EA 1/259/13). The study was conducted according to the common standard guidelines for clinical trials (Declaration of Helsinki and, where applicable, the International Conference on Harmonization of Technical Requirements for Registration of Pharmaceuticals for Human Use and Good Clinical Practice revised version, Somerset West, Republic of South Africa, 1996).

All study participants provided oral and written informed consent. The trial was registered at ClinicalTrials.gov (NCT02019134), and the study protocol has been published elsewhere [18].

## Results

### Baseline Characteristics

Of the 748 screened participants, 220 (29.4%) were eligible for the study and gave informed consent. They were randomized either to the app-based intervention group (110/220, 50%) or to the usual care group (110/220, 50%).

The sociodemographic and clinical characteristics of the participants at baseline are presented in Table 1. The participants had a mean age of 38.9 (SD 11.3) years and an average education, with 70% (154/220) having ≥10 years of school education. Of the 220 participants, 35 (15.9%) participants had a migration background. In the previous 7 days, the average neck pain on the NRS was 5.7 (SD 1.3) points, and 26.8% (59/220) of participants had taken medication for neck pain.

Although both groups were comparable at baseline, we observed small differences regarding gender (intervention vs control: female 74/110, 67.3% vs 79/110, 71.8%), partnership status (56/110, 50.9% vs 66/110, 60%), migration background (14/110, 12.7% vs 21/110, 19.1%), duration of neck pain (mean 79.2, SD 74.8 months vs mean 86.4, SD 97.7 months), and number of sick-leave days (mean 1.7, SD 3.6 days vs mean 2.1, SD 4.5 days) after randomization.

**Table 1:** Baseline demographic and clinical characteristics of the trial groups (N=220).

Characteristics			App-based intervention (n=110)		Control (n=110)
Age (years), mean (SD)			37.9 (11)		39.8 (11.6)
**Gender, n (%)**					
	Female	74 (67.3)		79 (71.8)	
	Male	36 (32.7)		31 (28.2)	
BMI (kg/m²), mean (SD)			24.5 (4.6)		23.9 (4.1)
Graduation after ≥10 years of school, n (%)			79 (71.8)		75 (68.2)
**Size of household, n (%)**					
	Single-person	32 (29.1)		34 (30.9)	
	2-person	44 (40)		42 (38.2)	
	Multiperson	34 (30.9)		34 (30.9)	
Partnership, n (%)			56 (50.9)		66 (60)
Migration background^a^, n (%)			14 (12.7)		21 (19.1)
Neck pain intensity in the previous 7 days (NRS^b,c^), mean (SD)			5.7 (1.4)		5.8 (1.3)
Neck pain–related stress intensity in the previous 7 days (NRS^c^), mean (SD)			5.4 (1.9)		5.3 (2.1)
Duration of neck pain (months), mean (SD)			79.2 (74.8)		86.4 (97.7)
Sick-leave days, mean (SD)			1.7 (3.6)		2.1 (4.5)
Medication intake against neck pain, n (%)			28 (25.5)		31 (28.2)
Pain acceptance, mean (SD)			73.3 (16.7)		73.6 (15.9)
Subscale pain willingness, mean (SD)			30.1 (10.1)		31.1 (8.2)
Subscale activity engagement, mean (SD)			43.2 (8.8)		42.4 (9)
**Expected effectiveness of relaxation exercise, n (%)**					
	Recovery	1 (0.9)		5 (4.5)	
	Distinct improvement	54 (49.1)		61 (55.5)	
	Light improvement	55 (50)		44 (40)	
	No improvement	0 (0)		0 (0)	
	Ineffective	0 (0)		0 (0)	
**Expected effectiveness of no relaxation exercise, n (%)**					
	Recovery	0 (0)		1 (0.9)	
	Distinct improvement	3 (2.7)		6 (5.5)	
	Light improvement	15 (13.6)		18 (16.4)	
	No improvement	89 (80.9)		81 (73.6)	
	Ineffective	3 (2.7)		4 (3.6)	

^a^Based on a study by Schenk et al [31].

^b^NRS: Numeric Rating Scale.

^c^Lower values indicate better status.

### Outcomes

Less intense mean neck pain was observed in both groups during the first 3 months compared with the baseline (Table 2). However, there was no significant difference in the primary outcome of the mean neck pain intensity during the first 3 months between the intervention and control groups (group difference 0.3, 95% CI –0.2 to 0.7; *P*=.23). In addition, no significant differences in the mean neck pain intensity between the 2 groups during the second 3 months (group difference −0.1, 95% CI −0.7 to 0.4; *P*=.62) or during the entire 6 months (group difference 0.1, 95% CI –0.3 to 0.6; *P*=.62) were found.

The subgroup analysis also yielded comparable primary outcomes between participants of different genders, ages, education levels, and disease durations.

**Table 2:** Primary and secondary outcomes (adjusted for sex and baseline value; N=220).

Outcome			App-based intervention, mean (95% CI)		Control, mean (95% CI)		Differences intervention versus control, mean (95% CI)		*P* value
Neck pain intensity during first 3 months (NRS^a,b^)			4.1 (3.8 to 4.4)		3.8 (3.5 to 4.1)		0.3 (−0.2 to 0.7)		.23
**Neck pain intensity (NRS^b^)**									
	Second 3 months	3.6 (3.2 to 4)		3.7 (3.4 to 4.1)		−0.1 (−0.7 to 0.4)		.62	
	First 6 months	3.9 (3.6 to 4.2)		3.8 (3.5 to 4.1)		0.1 (−0.3 to 0.6)		.62	
**Average neck pain during previous 7 days (NRS)**									
	First 3 months	4.3 (4 to 4.6)		4 (3.8 to 4.3)		0.2 (−0.2 to 0.7)		.24	
	Second 3 months	3.8 (3.4 to 4.1)		3.9 (3.6 to 4.3)		−0.2 (−0.7 to 0.3)		.52	
	First 6 months	4.1 (3.8 to 4.4)		4 (3.7 to 4.3)		0.2 (−0.3 to 0.6)		.49	
**Pain acceptance**									
	After 3rd month	75.4 (73 to 77.8)		75.8 (73.4 to 78.1)		−0.4 (−3.8 to 3)		.83	
	After 6th month	76.1 (73.7 to 78.4)		75.8 (73.6 to 78.1)		0.2 (−3 to 3.5)		.89	
**Participants with medication intake against neck pain, proportion (%)^c,d^**									
	During 6 months	49.5 (39.8 to 59.3)		52.4 (42.4 to 62.2)		0.97 (0.5 to 1.8)		.69	
**Numbers of weeks with pain medication**									
	First 3 months	2 (1.5 to 2.5)		2 (1.4 to 2.5)		0.01 (−0.7 to 0.8)		.98	
	Second 3 months	2 (1.4 to 2.6)		2 (1.5 to 2.6)		−0.03 (−0.8 to 0.8)		.93	
	First 6 months	3.7 (2.7 to 4.7)		3.9 (2.9 to 4.9)		−0.2 (−1.7 to 1.2)		.75	
**Neck pain–related stress**									
	First 3 months	4 (3.7 to 4.3)		3.8 (3.5 to 4.1)		0.2 (−0.2 to 0.7)		.32	
	Second 3 months	3.6 (3.2 to 3.9)		3.6 (3.2 to 4)		0 (−0.6 to 0.5)		.88	
	First 6 months	3.9 (3.6 to 4.2)		3.7 (3.4 to 4)		0.2 (−0.3 to 0.6)		.46	
**Responder rate, proportion (%)^c,d,e^**									
	After third month	29.4 (21 to 38.9)		35.6 (26.4 to 45.6)		0.75 (0.4 to 1.4)		.33	
	After sixth month	35.9 (26.8 to 45.7)		37.5 (28.2 to 47.5)		0.93 (0.5 to 1.7)		.80	
**Sick-leave days**									
	After third month	1.2 (0.4 to 2)		1.5 (0.7 to 2.3)		−0.3 (−1.4 to 0.9)		.66	
	After sixth month	1.1 (0.6 to 1.6)		1 (0.5 to 1.5)		0.1 (−0.6 to 0.8)		.81	
**Concomitant treatment, proportion (%)^c,d^**									
	After third month	40 (30.8 to 49.8)		45.5 (35.9 to 55.2)		0.82 (0.5 to 1.4)		.50	
	After sixth month	47.3 (37.7 to 57)		43.6 (34.2 to 53.4)		1.20 (0.7 to 2.1)		.69	

^a^NRS: Numeric Rating Scale.

^b^Lower values indicate better status.

^c^Between-group differences are presented as odds ratio (95% CI) instead of mean (95% CI).

^d^Proportions are not adjusted.

^e^Either at least 50% pain reduction or at least 2.5 points on the Numeric Rating Scale compared with baseline.

Furthermore, there were no significant differences between the mean average neck pain based on weekly measurements in either group during the first 3 months (group difference 0.2, 95% CI –0.2 to 0.7; *P*=.24), second 3 months (group difference –0.2, 95% CI –0.7 to 0.3; *P*=.52), or the entire 6 months (group difference 0.2, 95% CI –0.3 to 0.6; *P*=.49).

The chance of being a responder was similar for both groups after 3 months (odds ratio 0.75, 95% CI 0.4-1.4) and after 6 months (odds ratio 0.93, 95% CI 0.5-1.7).

There were also no significant differences in pain acceptance between the groups after 3 months (group difference −0.4, 95% CI −3.8 to 3; *P*=.83) and 6 months (group difference 0.2, 95% CI −3 to 3.5; *P*=.89).

There was no significant difference between the proportions of participants who took pain medication among both groups during the whole follow-up period of 6 months (odds ratio 0.97, 95% CI 0.5-1.8; *P*=.68). The number of weeks with pain medication did not differ between the groups in the first 3 months, second 3 months, and 6 months. The number of sick-leave days and pain acceptance did not differ between the groups.

The sensitivity and subgroup analyses did not change the pattern of the results, and we found no significant difference between female and male participants in a subgroup analysis of the primary outcome.

### App-Based Exercise Time and Study Dropout

**Figure 3:**
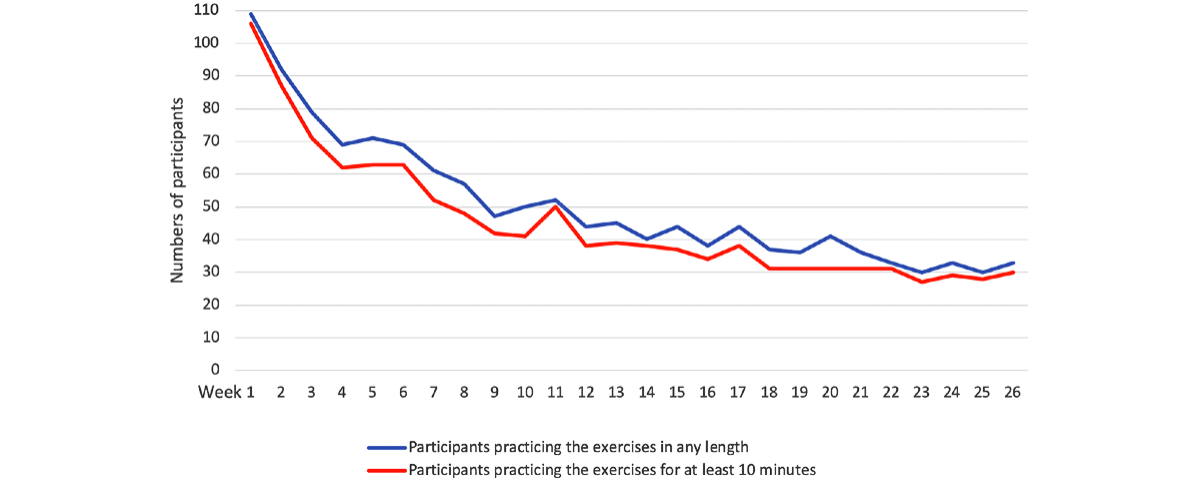
Number of participants practicing the exercises over time.

The overall time spent exercising declined with time. In the first week, almost all participants (109/110, 99.1%) in the intervention group practiced the exercises with the app. However, only 40% (44/110) of the participants continued to practice the exercises (for any length) in week 12, and 30% (33/110) of the participants continued to practice the exercises (for any length) in week 26. The declining trend was similar over the study phase when comparing the number of participants who practiced relaxation exercises of any length with the number of participants who practiced relaxation exercises for at least 10 minutes per week (Figure 3).

**Figure 4:**
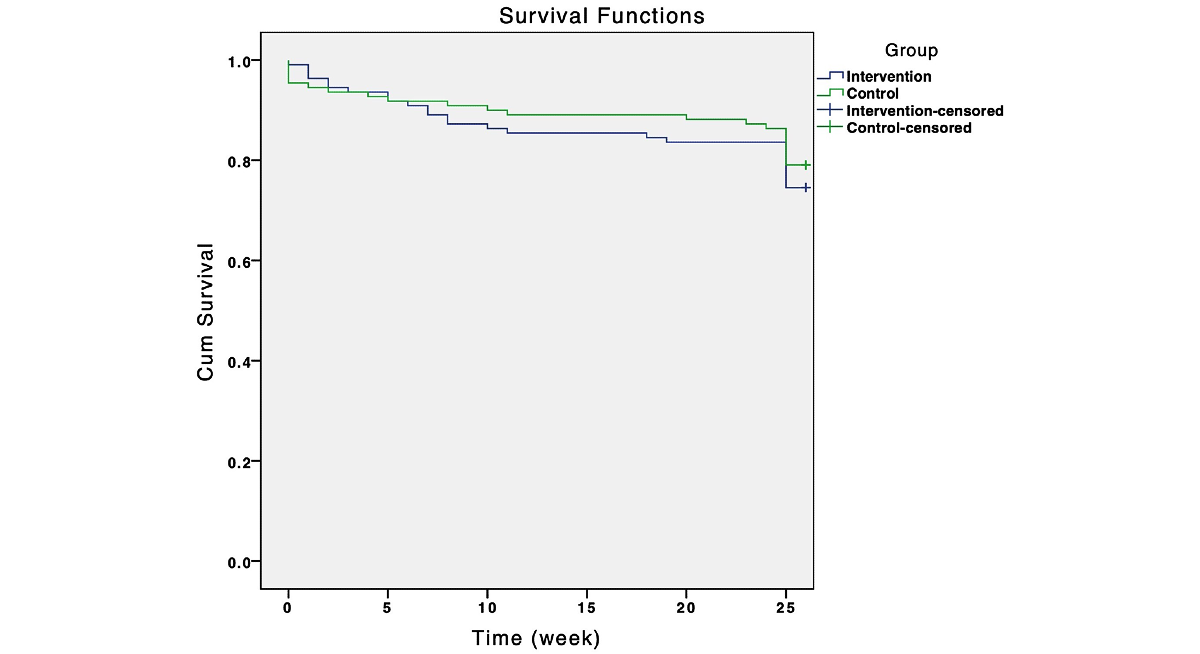
Probability of dropout in using the study app by group.

The Kaplan–Meier survival curves in Figure 4 display the study dropouts. There was no significant difference in the curves between the 2 groups according to the log-rank test (*P*=.44).

Approximately 74.5% (82/110) of participants in the intervention group and 79.1% (87/110) of participants in the control group used the study app to answer the survey questions until the end of the study (week 26).

### Self-perceived Neck Pain Change

**Figure 5:**
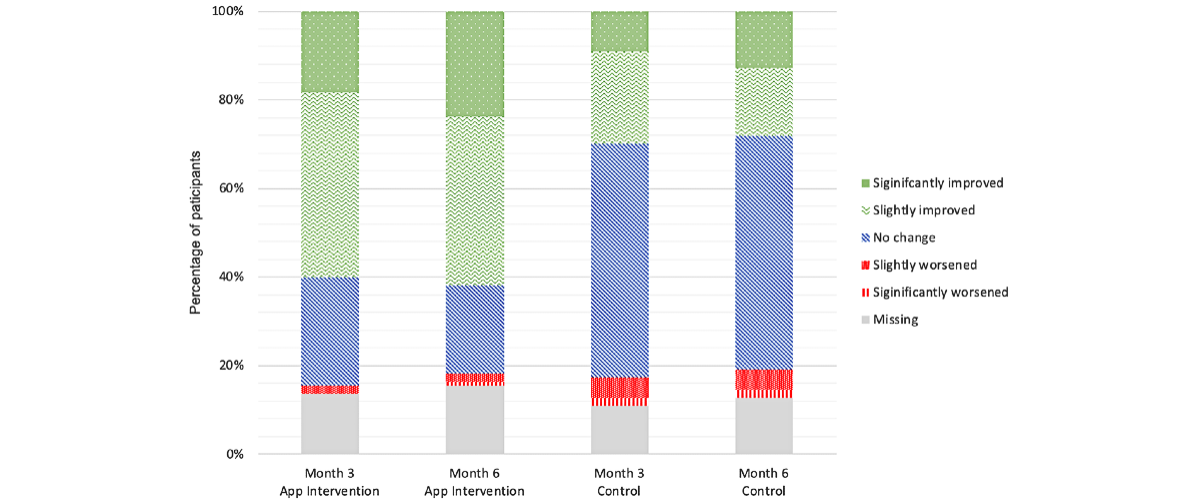
Self-perceived improvement of neck pain.

Overall, 60% (66/110) of participants in the intervention group reported that they felt the neck pain improved significantly or slightly after 3 and 6 months, in contrast to approximately 30% (33/110) of participants in the control group who said the same (Figure 5).

### BCTs in the App

Most parts of the app’s user interface implementations can be characterized as *prompt and cues* BCT, such as the dashboard dialog showing the number of questionnaires remaining to be processed. Moreover, the *prompt and cues* BCT was combined with the *action planning* BCT to remind participants to fill out their weekly diaries. Participants could determine the time and date of the reminders (action planning and prompt and cues BCT).

To ensure proper performance of the relaxation exercises, all the exercises were explained by experienced clinicians in an audio recording (*instruction on how to perform the behavior* BCT). The Relaxneck app provided the full name, profession, professional title, and workplace of the audio recording instructors to ensure quality and safety for the participants (*credible source* BCT).

### Safety Data

There were 5 serious adverse events recorded only in the control group, including cancer, sudden hearing loss, nerve injury and spinal tap, tonsillectomy, and an accident causing a fracture of the upper arm. None of them was considered related to the trial or the trial intervention.

## Discussion

### Principal Findings

In our trial, additional app-based self-relaxation techniques were not more effective than usual care alone for the reduction of chronic neck pain in a pragmatic setting. The results were consistent across all outcomes. The evaluated self-relaxation techniques were safe to use; however, they did not effectively relieve chronic neck pain during this app-based study.

There are a few possible reasons that helped to understand why the intervention did not improve pain. The study app’s design was not updated during the study (developed in 2014) and did not include more elaborate BCTs, such as feedback about the correct application of the intervention and monitoring [27]. As the retrospective BCT analysis showed, only *prompt and cues* BCT was mainly used, whereas modern digital interventions or consumer apps widely apply BCTs [32,33]. In mobile health settings, personalized feedback from the app would be a promising virtual communication tool to enhance patient engagement and adherence [34]. Biofeedback and self-monitoring of changes are very important in relaxation- and mindfulness-based therapies for pain. Moreover, it must be considered that our study mainly measured self-reported outcomes. The study may have benefited from parameters such as step count as a measure of physical activity or sleep duration as a proxy for sleep [35]. At the time when the study was planned, wearables were not widely implemented, and it was more difficult to link these measures with an app because of interoperability issues. However, the type and duration of the audio recordings used as interventions were measured and used as measures of adherence. Although tracked outcomes may have added a more objective point of view, the implementation would have added a much larger complexity during the development of the app. In addition, mindfulness-based therapies are very often designed with progressive lengths or difficulties [36]. In our trial, the participants were required to practice 3 relaxation exercises of almost the same length repeatedly across the whole intervention period. This could have limited the participants’ interest and the treatment effect. Finally, our app focused on audio relaxation alone instead of incorporating a whole theoretical framework such as mindfulness-based stress reduction or a comprehensive pain management strategy. Therefore, it is likely that the intervention of the study app was not powerful enough to improve chronic pain.

Adherence to the trial intervention was low compared with other app-based studies conducted by our research group [30,37]. The number of participants who performed the relaxation exercises diminished during the course of the study. Potential explanations may again be the lack of an elaborate BCT concept or that chronic pain decreases motivation [38], especially to perform prescribed physical activities and exercises [39]. However, the number of practiced exercises of any length or >10 minutes remained similar over time. This might indicate that users who feel attached to the app-based relaxation exercise at the beginning finish the whole exercise process in most cases.

Although our study intervention was asynchronous, that is, contact with a health care provider and app intervention occurred at different time points, future mobile health studies may also include synchronous interventions in which health care providers could offer real-time interventions to the users. This approach might be helpful to improve the app and study adherence. However, this approach might also increase the complexity of the intervention and increase the costs.

In our trial, stopping the app-based intervention did not necessarily predict stopping the answering of the app-based survey questions. Only 30% (33/110) of the participants continued to practice the app-based relaxation exercises until the end of the follow-up; however, 74.5% (82/110) of participants used the app to answer survey questions until the end of the trial. Meanwhile, adherence to app use for answering survey questions was not affected by whether the app contained intervention features. The proportion of participants who used the app regularly to answer surveys until the end of the study was rather similar in both groups. A possible explanation for the good response rate in both groups could be our reminder system for the questionnaires or the paid compensation for the efforts.

Although all other outcomes did not show statistically significant group differences, most participants in the intervention group reported self-perceived improvement of neck pain, whereas most participants in the control group reported no change or worsening of neck pain. This result might be attributed to a digital placebo effect. The concept of the digital placebo effect has already been discussed in mental health studies [40]. A good example could be seen in a study involving a smartphone app that was designed to help patients self-monitor and record their symptoms of depression. Even without any direct therapeutic intervention, smartphone-based self-monitoring significantly reduced the symptoms [41]. Future studies should investigate the perceived changes in pain and the placebo-like effects of smartphone interventions.

### Strengths and Limitations

Our app-based RCT was performed in a pragmatic setting. In addition, stakeholder engagement was implemented in the design of the trial and intervention [18]. Hence, the selection of the relaxation exercises and the length of the exercises were defined during stakeholder meetings to facilitate patient-centered therapy. Moreover, the study included a sufficient number of participants to answer our research question. Thus, our findings were considered generalizable in a real-life setting.

Some limitations have to be considered for this trial. The trial recruitment took rather long (32 months), possibly because of our conventional on-site recruitment strategy with paper-and-pencil baseline questionnaires. During that time, smartphone technologies, designs, and perceptions experienced numerous changes. For example, it is unclear whether the app’s user interface was perceived as outdated by the participants. For future app-based studies, web-based recruitment and the incorporation of an app-based baseline survey could accelerate the overall trial process [15]. This acceleration of the trial process might also increase the relevance of the results.

Potential selection bias with an impact on the generalizability of the results might be another limitation of this study. The trial was conducted from 2014 to 2017. All study participants needed to own a smartphone. However, at that time, the number of smartphone owners in Germany (approximately 50%) was substantially lower than the current number (approximately 72%) [42]. It is unclear whether this affected the characteristics of our study population. To address a broader user base, we decided to build the study app for both the main platforms (iOS and Android).

Unfortunately, our sample size could not enable gender disaggregation. Gender might influence behavioral change, use patterns, and adherence to app use [43]. Some app-based studies have reported that gender is a strong predictor of the discontinuation of relaxation app use [37,44]. In this study, approximately 69.5% (153/220) of the participants were women. It would be interesting to discover the role of sex and gender in participants’ adherence in future studies.

During the development of the app, we did not follow a preplanned BCT concept, and only basic BCTs were implemented, as shown in the post hoc review of the BCT techniques used. However, regarding behavioral change and intervention effects, a meta-analysis [45] concluded that implementing more (than one) theory is unlikely to improve intervention effectiveness. Future studies should be conducted to better understand the impact of BCTs on intervention outcomes for interventions for chronic pain.

Finally, the trial was single-blinded, as we could not blind the participants. However, it is common that participants cannot be blinded in nonpharmacological complex intervention trials and eHealth trials.

### Comparison With Previous Work

Mind–body therapies are considered to be relatively safe [46]. However, only a few studies have been conducted on chronic neck pain. There were not enough trials for the Institute for Clinical and Economic Review (ICER) to summarize the effectiveness of cognitive and mind–body therapies for chronic neck pain [14]. According to a systematic review that investigated the effects of mindfulness- and relaxation-based interventions in an eHealth setting [47], only a few studies reported positive effects on pain, and no study reported positive effects on stress or mindfulness.

However, some eHealth studies have been conducted for chronic lower back pain. Heapy et al [48] reported that the efficacy of cognitive behavioral therapies (CBTs) delivered remotely using telephone and the internet for chronic back pain is not inferior to that of in-person CBTs. Kristjánsdóttir et al [49] reported that smartphone app–based interventions with personalized feedback can reduce catastrophizing in women with chronic widespread pain. Instead of relaxation exercises alone, CBT, including emotion recognition, mindfulness exercises, and empathic communication, was highlighted in these studies. It seems that the evidence for only relaxation is rather low compared with systematic mind–body therapy or CBT for chronic pain. Therefore, future studies are required to investigate the effect of mind–body therapy on chronic neck pain within a comprehensive pain management strategy.

### Conclusions

In conclusion, the evaluated study smartphone app, which included self-relaxation techniques such as autogenic training, mindfulness meditation, and guided imagery but without elaborate BCTs, was not more effective than usual care for chronic neck pain in a pragmatic trial. Further studies are needed to understand the potential of relaxation for neck pain and whether app-based mechanisms for relaxation and behavior change might be useful within a comprehensive pain management strategy for neck pain.

## Appendix

Multimedia Appendix 1 CONSORT-eHEALTH checklist (V 1.6.2).

## Abbreviations

BCT: behavior change technique
CBT: cognitive behavioral therapy
ICER: Institute for Clinical and Economic Review
NRS: Numeric Rating Scale
RCT: randomized controlled trial
