# Supplementary material for: App-Based Relaxation Exercises for Patients With Chronic Neck Pain: Pragmatic Randomized Trial
Source: JMIR Mhealth Uhealth. 2022 Jan 7;10(1):e31482. doi: 10.2196/31482 (PMC8783271; doi:10.2196/31482)
Supplement: Multimedia Appendix 1 [file mhealth_v10i1e31482_app1.pdf]

CONSORT-EHEALTH Checklist V1.6.2 Report

	Manuscript Number	31482
**(based on CONSORT-EHEALTH V1.6), available at [http://tinyurl.com/consort-ehealth-v1-6].**		

Date completed
12/23/2021 19:06:54
by
Daniel Pach

TITLE

1a-i) Identify the mode of delivery in the title

1a-ii) Non-web-based components or important co-interventions in title
App-Based Relaxation Exercises for Patients With Chronic Neck Pain: Pragmatic Randomized Trial
1a-iii) Primary condition or target group in the title

ABSTRACT

1b-i) Key features/functionalities/components of the intervention and comparator in the METHODS section of the ABSTRACT

1b-ii) Level of human involvement in the METHODS section of the ABSTRACT
these items were not presented in the abstract
1b-iii) Open vs. closed, web-based (self-assessment) vs. face-to-face assessments in the METHODS section of the ABSTRACT

1b-iv) RESULTS section in abstract must contain use data

1b-v) CONCLUSIONS/DISCUSSION in abstract for negative trials
"In week 12, only 40% (44/110) of the participants in the intervention group continued to practice the exercises with the app."

INTRODUCTION

2a-i) Problem and the type of system/solution

2a-ii) Scientific background, rationale: What is known about the (type of) system
Moreover, according to the recent Neck Pain Guideline of the German Society of General Practice and Family Medicine [13], learning a relaxation technique is recommended for patients with nonspecific chronic neck pain that lasts for >12 weeks. Thus, relaxation techniques alone or in addition to conventional medical care can influence the treatment and rehabilitation of chronic neck pain. However, the accessibility of cognitive and mind–body therapies for chronic low back pain and neck pain remains a major challenge [14].

METHODS

3a) CONSORT: Description of trial design (such as parallel, factorial) including allocation ratio

3b) CONSORT: Important changes to methods after trial commencement (such as eligibility criteria), with reasons
"In this study, we aim to conduct a pragmatic app-based RCT to evaluate whether app-based audio relaxation exercises are more effective in reducing chronic neck pain than usual care."
3b-i) Bug fixes, Downtimes, Content Changes

4a) CONSORT: Eligibility criteria for participants
App-Based Relaxation Exercises for Patients With Chronic Neck Pain: Pragmatic Randomized Trial
4a-i) Computer / Internet literacy

4a-ii) Open vs. closed, web-based vs. face-to-face assessments:
possession of a smartphone (iOS or Android) was an inclusion criteria, computer literacy was not
4a-iii) Information giving during recruitment
"Eligibility was checked by a study nurse at the study site. Eligible participants completed the paper-and-pencil baseline questionnaires. Then, the study nurse helped the participants install the app on their own smartphones and provided a randomly allocated code to activate the study app and the respective app features..."
4b) CONSORT: Settings and locations where the data were collected
there were no changes after trial commencement
4b-i) Report if outcomes were (self-)assessed through online questionnaires

4b-ii) Report how institutional affiliations are displayed
All study data after baseline measurements were collected by means of app-based diaries and questionnaires."
5) CONSORT: Describe the interventions for each group with sufficient details to allow replication, including how and when they were actually administered
5-i) Mention names, credential, affiliations of the developers, sponsors, and owners

5-ii) Describe the history/development process
"The study app Relaxneck was developed by the Institute for Social Medicine, Epidemiology, and Health Economics, Charité–Universitätsmedizin Berlin, Germany, together with Smart Mobile Factory, Berlin, Germany, which is an agency focused on mobile solutions [18]."
5-iii) Revisions and updating
development not addressed in detail
5-iv) Quality assurance methods
this subitem was not applicable
5-v) Ensure replicability by publishing the source code, and/or providing screenshots/screen-capture video, and/or providing flowcharts of the algorithms used
we have not included this subitem
5-vi) Digital preservation
we included screenshots in the manuscript
5-vii) Access
we did not address this subitem
5-viii) Mode of delivery, features/functionalities/components of the intervention and comparator, and the theoretical framework
"Information on the study was posted with brochures and posters in universities, gyms, and general practitioners' offices. Moreover, the study was advertised in local subways from December 2014 to July 2015. Eligibility was checked by a study nurse at the study site. Eligible participants completed the paper-and-pencil baseline questionnaires. Then, the study nurse helped the participants install the app on their own smartphones and provided a randomly allocated code to activate the study app and the respective app features according to the group allocation..."
5-ix) Describe use parameters
"There were 3 types of exercises (autogenic training, mindfulness meditation, and guided imagery), with a length of 15 minutes each, that were available in 2 versions (female and male voices)..."
5-x) Clarify the level of human involvement
"It was recommended to apply a relaxation exercise daily or at least 5 days per week for 6 months."

**5-xi) Report any prompts/reminders used**		
"Eligibility was checked by a study nurse at the study site. Eligible participants completed the paper-and-pencil baseline questionnaires. Then, the study nurse helped the participants install the app on their own smartphones and provided a randomly allocated code to activate the study app..." "if 2 consecutive weekly surveys had not been completed, the patient was contacted by telephone call"		
**5-xii) Describe any co-interventions (incl. training/support)**		
"If a weekly survey had not been completed, the patient received an SMS text message as a reminder; if 2 consecutive weekly surveys had not been completed, the patient was contacted by telephone call; if there was no response after 2 calls, the patient received a reminder letter."		
**6a) CONSORT: Completely defined pre-specified primary and secondary outcome measures, including how and when they were assessed**		
"The inclusion criteria were as follows: aged 18-65 years, chronic neck pain within at least the past 12 weeks, average neck pain intensity ≥4 on the Numeric Rating Scale (NRS; 0=no pain to 10=worst possible pain) in the previous week, possession of a smartphone (iOS or Android), willingness to be randomized and follow the app-delivered interventions, and willingness to enter data through the study app..."		
**6a-i) Online questionnaires: describe if they were validated for online use and apply CHERRIES items to describe how the questionnaires were designed/deployed**		
**6a-ii) Describe whether and how "use" (including intensity of use/dosage) was defined/measured/monitored**		
this item is not applicable		
**6a-iii) Describe whether, how, and when qualitative feedback from participants was obtained**		
The number of participants who practiced the exercises was recorded to reflect exercise adherence over time. Practice of the exercise was defined by (1) tracking the number (and duration) of applied types of intervention with the app and (2) asking the participants weekly about the number of applied types of intervention without using the app. The complete stop of filling in any data with the study app was defined as participant dropout.		
**6b) CONSORT: Any changes to trial outcomes after the trial commenced, with reasons**		
"Eligible participants completed the paper-and-pencil baseline questionnaires..."		
**7a) CONSORT: How sample size was determined**		
**7a-i) Describe whether and how expected attrition was taken into account when calculating the sample size**		
**7b) CONSORT: When applicable, explanation of any interim analyses and stopping guidelines**		
"The primary outcome measure was the mean neck pain intensity during the first 3 months of intervention based on daily measurements of pain intensity on the NRS (0=no pain to 10=worst possible pain) [18].<br>The secondary outcome parameters included the mean pain intensity during the first 6 months after randomization based on daily measurements, the mean pain intensity measured weekly (using NRS) as the average pain intensity of the previous 7 days over 3 and 6 months, pain acceptance..."		
**8a) CONSORT: Method used to generate the random allocation sequence**		
this item was not applicable		
**8b) CONSORT: Type of randomisation; details of any restriction (such as blocking and block size)**		
this item was not applicable		
**9) CONSORT: Mechanism used to implement the random allocation sequence (such as sequentially numbered containers), describing any steps taken to conceal the sequence until interventions were assigned**		
"The randomization sequence was generated by a data manager who was not involved in the analysis of the data or the enrollment of the patients; SAS (version 9.3, SAS Inc) was used for this process. The randomization list was included in a safe Microsoft Access database to ensure that it was not accessible during the randomization process of individual participants and that the screened patients were strictly consecutively enrolled. The randomization process was conducted by the study office at the Institute for Social Medicine, Epidemiology, and Health Economics. To ensure allocation concealment, first, the study team added the participants' information into the database, and then, random allocation of the participants into the intervention or control group was performed."		
**10) CONSORT: Who generated the random allocation sequence, who enrolled participants, and who assigned participants to interventions**		
"using blocked randomization with variable block lengths and an allocation ratio of 1:1, that is, 110:110 participants."		
**11a) CONSORT: Blinding - If done, who was blinded after assignment to interventions (for example, participants, care providers, those assessing outcomes) and how**		
**11a-i) Specify who was blinded, and who wasn't**		
**11a-ii) Discuss e.g., whether participants knew which intervention was the "intervention of interest" and which one was the "comparator"**		
Finally, the trial was single-blinded, as we could not blind the participants. However, it is common that participants cannot be blinded		
**11b) CONSORT: If relevant, description of the similarity of interventions**		
"The randomization sequence was generated by a data manager who was not involved in the analysis of the data or the enrollment of the patients; SAS (version 9.3, SAS Inc) was used for this process. The randomization list was included in a safe Microsoft Access database to ensure that it was not accessible during the randomization process of individual participants and that the screened patients were strictly consecutively enrolled. The randomization process was conducted by the study office at the Institute for Social Medicine, Epidemiology, and Health Economics. To ensure allocation concealment, first, the study team added the participants' information into the database, and then, random allocation of the participants into the intervention or control group was performed."		
**12a) CONSORT: Statistical methods used to compare groups for primary and secondary outcomes**		
a protocol publication is available		
**12a-i) Imputation techniques to deal with attrition / missing values**		
**12b) CONSORT: Methods for additional analyses, such as subgroup analyses and adjusted analyses**		
this was not specifically discussed in the manuscript		
RESULTS		
**13a) CONSORT: For each group, the numbers of participants who were randomly assigned, received intended treatment, and were analysed for the primary outcome**		
**13b) CONSORT: For each group, losses and exclusions after randomisation, together with reasons**		
"For sensitivity analysis, the primary analysis of the primary outcome was repeated based on the per-protocol population.<br>Subgroup analyses were performed on the primary outcome by including an interaction term (subgroup variable by treatment) in the main model and performing separate analyses for each subgroup. Subgroups were specified with covariates in age, education (>10 years of school education or ≤10 years of school education), sex (male or female), and duration of disease. Kaplan–Meier survival analysis was conducted to investigate whether the app features (with or without app-based intervention content) predicted the dropout of app use."		
**13b-i) Attrition diagram**		
**14a) CONSORT: Dates defining the periods of recruitment and follow-up**		
this is shown in the manuscript		
**14a-i) Indicate if critical "secular events" fell into the study period**		
**14b) CONSORT: Why the trial ended or was stopped (early)**		
this is shown in the flow chart		
**15) CONSORT: A table showing baseline demographic and clinical characteristics for each group**		
"The first participant was randomized on March 31, 2014, and the final data recording was on January 11, 2017, in Berlin, Germany. "		
**15-i) Report demographics associated with digital divide issues**		
**16a) CONSORT: For each group, number of participants (denominator) included in each analysis and whether the analysis was by original assigned groups**		
**16-i) Report multiple "denominators" and provide definitions**		
**16-ii) Primary analysis should be intent-to-treat**		

this is transparently shown		
**17a) CONSORT: For each primary and secondary outcome, results for each group, and the estimated effect size and its precision (such as 95% confidence interval)**		
long recruitment period is discussed		
**17a-i) Presentation of process outcomes such as metrics of use and intensity of use**		
**17b) CONSORT: For binary outcomes, presentation of both absolute and relative effect sizes is recommended**		
a table with baseline demographic and clinical characteristics for each group is shown		
**18) CONSORT: Results of any other analyses performed, including subgroup analyses and adjusted analyses, distinguishing pre-specified from exploratory**		
results are shown accordingly		
**18-i) Subgroup analysis of comparing only users**		
**19) CONSORT: All important harms or unintended effects in each group**		
this item is not applicable		
**19-i) Include privacy breaches, technical problems**		
**19-ii) Include qualitative feedback from participants or observations from staff/researchers**		
this was not applicable and therefore not mentioned in the manuscript		
DISCUSSION		
**20) CONSORT: Trial limitations, addressing sources of potential bias, imprecision, multiplicity of analyses**		
**20-i) Typical limitations in ehealth trials**		
**21) CONSORT: Generalisability (external validity, applicability) of the trial findings**		
**21-i) Generalizability to other populations**		
**21-ii) Discuss if there were elements in the RCT that would be different in a routine application setting**		
applicability of the findings are discussed		
**22) CONSORT: Interpretation consistent with results, balancing benefits and harms, and considering other relevant evidence**		
**22-i) Restate study questions and summarize the answers suggested by the data, starting with primary outcomes and process outcomes (use)**		
**22-ii) Highlight unanswered new questions, suggest future research**		
we restated the study questions and summarized the answers		
Other information		
**23) CONSORT: Registration number and name of trial registry**		
"The sensitivity and subgroup analyses did not change the pattern of the results, and we found no significant difference between female and male participants in a subgroup analysis of the primary outcome."		
**24) CONSORT: Where the full trial protocol can be accessed, if available**		
safety data was reported in the manuscript		
**25) CONSORT: Sources of funding and other support (such as supply of drugs), role of funders**		
registrations numbers were presented		
**X26-i) Comment on ethics committee approval**		
**x26-ii) Outline informed consent procedures**		
"The study was approved by the local ethics review board at the Charité–Universitätsmedizin, Berlin (approval number Relaxneck EA 1/259/13). "		
**X26-iii) Safety and security procedures**		
"Information on the study was posted with brochures and posters in universities, gyms, and general practitioners' offices. Moreover, the study was advertised in local subways from December 2014 to July 2015. Eligibility was checked by a study nurse at the study site. Eligible participants completed the paper-and-pencil baseline questionnaires. "		
**X27-i) State the relation of the study team towards the system being evaluated**		
